# Twins in spirit - episode I: comparative preclinical evaluation of [^68^Ga]DOTATATE and [^68^Ga]HA-DOTATATE

**DOI:** 10.1186/s13550-015-0099-x

**Published:** 2015-04-10

**Authors:** Margret Schottelius, Jakub Šimeček, Frauke Hoffmann, Marina Willibald, Markus Schwaiger, Hans-Jürgen Wester

**Affiliations:** Pharmaceutical Radiochemistry, Technical University Munich, Walther-Meissner-Strasse 3, 85748 Garching, Germany; Department of Nuclear Medicine, Klinikum rechts der Isar, Technical University Munich, Ismaningerstr. 22, 81675 Munich, Germany

**Keywords:** Somatostatin receptor, sst, Octreotate, DOTATATE, HA-DOTATATE, ^68^Ga, ^177^Lu, PET, PRRT

## Abstract

**Background:**

Recently, an intra-patient comparison demonstrated that the somatostatin (sst) ligand [^68^Ga]HA-DOTATATE ([^68^Ga]DOTA-3-iodo-Tyr^3^-octreotate) provides PET images comparable to or superior to those obtained with [^68^Ga]DOTATATE. To provide a comprehensive basis for nevertheless observed slight differences in tracer biodistribution and dosimetry, the characteristics of [^68^Ga]HA-DOTATATE were investigated in a detailed preclinical study.

**Methods:**

Affinities of ^nat^Ga-HA-DOTATATE and ^nat^Ga-DOTATATE to sst_1–5_ were determined using membrane preparations and [^125^I]SST-28 as radioligand. Internalization into AR42J cells was studied in dual-tracer studies with [^125^I]TOC as internal reference. Biodistribution was investigated using AR42J tumor-bearing CD1 mice, and specificity of tracer uptake was confirmed in competition studies by coinjection of 0.8 mg TOC/kg.

**Results:**

Sst_2_ affinities (IC_50_) of [^nat^Ga]HA-DOTATATE (1.4 ± 0.8 nM, log*P*: −3.16) and [^nat^Ga]DOTATATE (1.2 ± 0.6 nM, log*P*: −3.69) were nearly identical. Both compounds displayed IC_50_ > 1 μM for sst_1,3,4_, while sst_5_ affinity was markedly increased for ^nat^Ga-HA-DOTATATE (102 ± 65 nM vs >1 μM for ^nat^Ga-DOTATATE). [^nat^Lu]HA-DOTATATE and [^nat^Lu]DOTATATE showed slightly lower, identical sst_2_ affinities (2.0 ± 1.6 and 2.0 ± 0.8 nM, respectively) and sst_3_ affinities of 93 ± 1 and 162 ± 16 nM. Internalization of [^68^Ga]HA-DOTATATE was tenfold higher than that of [^125^I]TOC but only sixfold higher for [^68^Ga]DOTATATE and [^177^Lu]HA-DOTATATE. While [^68^Ga]HA-DOTATATE and [^68^Ga]DOTATATE had shown similar target- and non-target uptake in patients, biodistribution studies in mice at 1 h post injection (*n* = 5) revealed slightly increased non-specific uptake of [^68^Ga]HA-DOTATATE in the blood, liver, and intestines (0.7 ± 0.3, 1.0 ± 0.2, and 4.0 ± 0.7 %iD/g vs 0.3 ± 0.1, 0.5 ± 0.1, and 2.7 ± 0.8 %iD/g for [^68^Ga]DOTATATE). However, sst-mediated accumulation of [^68^Ga]HA-DOTATATE in the pancreas, adrenals, and tumor was significantly enhanced (36.6 ± 4.3, 10.8 ± 3.2, and 33.6 ± 10.9 %iD/g vs 26.1 ± 5.0, 5.1 ± 1.4, and 24.1 ± 4.9 %iD/g, respectively). Consequently, tumor/background ratios for [^68^Ga]HA-DOTATATE in the AR42J model are comparable or slightly increased compared to [^68^Ga]DOTATATE.

**Conclusions:**

The present preclinical data fully confirm the general biodistribution pattern and excellent *in vivo* sst-targeting characteristics previously observed for [^68^Ga]HA-DOTATATE in patients. The effect of slightly enhanced lipophilicity on background accumulation and normal organ dose is compensated by the high uptake of [^68^Ga]HA-DOTATATE in tumor. Thus, [^68^Ga]HA-DOTATATE represents a fully adequate, freely available substitute for [^68^Ga]DOTATATE and, given the superb sst-targeting characteristics of [^177^Lu]HA-DOTATATE *in vitro*, potential applicability for sst-targeted PRRT.

## Background

The excellent suitability of ^68^Ga-labeled octreotide analogs for functional imaging of somatostatin (sst) receptor-overexpressing tumors is well documented throughout the literature and has been thoroughly reviewed in recent publications [1,2]. Both [^68^Ga]DOTATOC- ([^68^Ga]DOTA-Tyr^3^-octreotide) and [^68^Ga]DOTATATE- ([^68^Ga]DOTA-Tyr^3^-octreotate) PET have been established as the imaging modality of choice for the diagnosis and management of patients with neuroendocrine tumors (NETs). Of the two tracers, [^68^Ga]DOTATATE shows slight advantages with respect to normal organ distribution, while tumor detection rates are identical to those of [^68^Ga]DOTATOC [3]. Recently, [^68^Ga]DOTANOC ([^68^Ga]DOTA-Nal^3^-octreotide) has also entered the clinical arena [4]. Due to its high affinity to sst_2_, sst_3_ and sst_5_, significantly more and smaller lesions are detected using [^68^Ga]DOTANOC compared to the sst_2_-selective high-affinity ligand [^68^Ga]DOTATATE, suggesting it to be the preferred agent for the imaging application [5].

However, in the context of sst-targeted endoradiotherapy using the respective ^177^Lu- or ^90^Y-labeled peptides, [^177^Lu]DOTATATE compared favorably to [^177^Lu]DOTANOC [6]. Due to its high sst_2_ selectivity, [^177^Lu]DOTATATE showed significantly lower uptake/dose delivered to normal tissues, while there were no significant differences concerning tumor kinetics and mean absorbed tumor dose. Thus, in the context of patient selection for sst-targeted endoradiotherapy with [^177^Lu]DOTATATE, [^68^Ga]DOTATATE-PET represents the imaging strategy of choice for an accurate assessment of sst expression levels.

**Figure 1:**
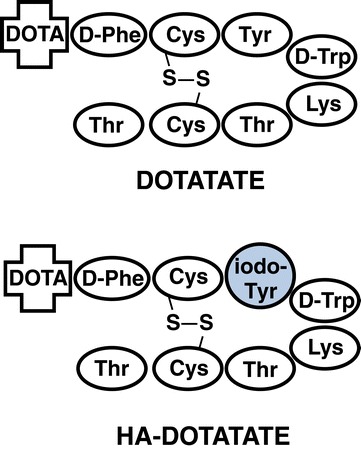
**Schematic representation of the structures of DOTATATE and HA-DOTATATE.**

Unfortunately, the availability of DOTATATE is governed by patent restrictions. With the intent to provide a suitable alternative with unchallenged *in vivo* performance, we recently introduced [^68^Ga]HA-DOTATATE ([^68^Ga]high-affinity DOTATATE, i.e., [^68^Ga]DOTA-3-iodo-Tyr^3^-octreotate, Figure 1). In the first *in vitro* studies, Ga-HA-DOTATATE had shown unchangedly high sst_2_ affinity compared to Ga-DOTATATE and increased affinity to sst_5_ [7]. Given that the physicochemical properties of [^68^Ga]HA-DOTATATE are very similar to those of [^68^Ga]DOTATATE, we directly transferred [^68^Ga]HA-DOTATATE into the first patients. Intra-patient comparison of [^68^Ga]DOTATATE- and [^68^Ga]HA-DOTATATE-PET revealed slightly enhanced uptake of the high-affinity analog in sst-expressing tissues as well as a marginally increased accumulation in the excretion organs. These characteristics lead to a remarkably uniform performance of [^68^Ga]DOTATATE and [^68^Ga]HA-DOTATATE in PET imaging of sst-expressing NETs [8]. However, not unexpectedly, the observed slight increase in specific and non-specific tissue accumulation of [^68^Ga]HA-DOTATATE leads to somewhat higher organ doses compared to [^68^Ga]DOTATATE [9]. To provide a basis for understanding the underlying reasons for these differences and thus for the adequate interpretation of our observations in patients, we performed a detailed preclinical evaluation of [^68^Ga]HA-DOTATATE in comparison to [^68^Ga]DOTATATE. Envisaging its potential use in peptide receptor radiotherapy, [^177^Lu]HA-DOTATATE was also included in the *in vitro* evaluation for a first comparative assessment of its binding and internalization characteristics.

## Methods

### Peptide synthesis

#### General conditions

2-CTC (2-chlorotrityl chloride) resin, coupling reagents as well as most Fmoc amino acids were obtained from Iris Biotech (Marktredwitz, Germany). Fmoc-3-iodo-Tyr and (Leu^8^,D-Trp^22^,[^125^I]Tyr^25^)-somatostatin-28 were supplied by Bachem (Heidelberg, Germany). All other organic reagents were purchased from VWR (Darmstadt, Germany) or Sigma-Aldrich or Fluka (Munich, Germany). Solvents were used without further purification. Solid phase peptide synthesis was carried out manually using an Intelli-Mixer syringe shaker (Neolab, Heidelberg, Germany).

Analytic RP-HPLC was performed on a Nucleosil 100 C18 (5 μm, 125 × 4.0 mm internal diameter (i.d.)) column (CS GmbH, Langerwehe, Germany) using a Sykam gradient HPLC System (Sykam, Fürstenfeldbruck, Germany).

The peptides were eluted applying various gradients of 0.1% TFA (trifluoroacetic acid) in H_2_O (solvent A) and 0.1% TFA in acetonitrile (solvent B) over 15 min at a constant flow of 1 mL/min. Preparative RP-HPLC was performed on the same HPLC system using a Multospher 100 RP 18–5 (250 × 10 mm i.d.) column (CS GmbH, Langerwehe, Germany) at a constant flow of 5 mL/min. UV detection was performed at 220 nm using a 206 PHD UV–vis detector (Linear™ Instruments Corporation, Reno, NV, USA). For radioactivity measurement, the outlet of the UV photometer was connected to a NaI(Tl) well-type scintillation counter from EG&G Ortec (München, Germany). Radio-TLC was carried out using a Varian silica impregnated ITLC chromatography paper (Varian Inc., Palo Alto, CA, USA) and a 1:1 (*v*/*v*) mixture of 1 M aq. NH_4_OAc and MeOH as mobile phase. TLC strips were analyzed using a miniGita TLC analyzer (Raytest, Straubenhardt, Germany).

Mass spectra were recorded on the LC-MS system LCQ from Finnigan (Bremen, Germany) using a Hewlett Packard series 1100 HPLC system (Agilent Technologies Inc., Santa Clara, CA, USA).

#### DOTATATE and HA-DOTATATE

DOTATATE and HA-DOTATATE were synthesized according to a previously published protocol [10]. Briefly, the sequences H_2_N-d-Phe-Cys(Trt)-Tyr(tBu)-d-Trp-Lys(Dde)-Thr(tBu)-Cys(Trt)-Thr(tBu)-OH (DOTATATE) and H_2_N-d-Phe-Cys(Trt)-3-iodo-Tyr-d-Trp-Lys(Dde)-Thr(tBu)-Cys(Trt)-Thr(tBu)-OH (HA-DOTATATE) were assembled on 2-CTC resin using a standard Fmoc protocol. After cleavage from the solid support using TFA/H_2_O/TIBS (triisobutylsilane) (95/2.5/2.5 (*v*/*v*/*v*)), the Lys^5^(Dde)-protected peptides were cyclized using H_2_O_2_ in an aqueous THF (tetrahydrofurane) solution at pH 7. DOTA coupling was carried out in aqueous DMF (dimethylformamide) solution using unprotected DOTA and NHS and EDCI as coupling reagents. Subsequent removal of the Lys^5^(Dde) protecting group was carried out using 2% of hydrazine hydrate in DMF. Crude peptides were then purified via preparative HPLC.

DOTATATE (C_65_H_90_N_14_O_19_S_2_): calculated monoisotopic mass = 1,434.6

Found: *m/z* = 1,436.1 [M + H]^+^, *m/z* = 1,458.0 [M + Na]^+^, 718.8 [M + 2H]^2+^

HA-DOTATATE (C_65_H_89_N_14_IO_19_S_2_): calculated monoisotopic mass = 1,560.5

Found: *m/z* = 1,561.6 [M + H]^+^, *m/z* = 1,583.5 [M + Na]^+^

#### Synthesis of ^nat^Ga, ^nat^Lu, and ^nat^Y reference compounds

DOTATATE and HA-DOTATATE (200 to 500 μg) were dissolved in 20 mM GaNO_3_, LuCl_3_, or YCl_3_ solution (in 0.01 M HCl), respectively, to yield a final peptide concentration of 1 mM. After heating to 95°C for 30 min in a sealed tube, HPLC analysis revealed quantitative complex formation without side products. The product solutions were used as such for the preparation of dilution series for binding studies.

^*nat*^*Ga-DOTATATE* (C_65_H_88_N_14_O_19_S_2_Ga): calculated monoisotopic mass = 1,501.6

Found: *m/z* = 752.6 [M + 2H]^2+^

^*nat*^*Ga-HA-DOTATATE* (C_65_H_87_N_14_IO_19_S_2_Ga): calculated monoisotopic mass = 1,628.5

Found: *m/z* = 1,629.1 [M + H]^+^, 1,651.0 [M + Na]^+^, 814.2 [M + 2H]^2+^

^*nat*^*Lu-DOTATATE* (C_65_H_87_N_14_O_19_S_2_Lu): calculated monoisotopic mass = 1,606.6

Found: *m/z* = 804.6 [M + 2H]^2+^

^*nat*^*Lu-HA-DOTATATE* (C_65_H_86_N_14_IO_19_S_2_Lu): calculated monoisotopic mass = 1,732.5

Found: *m/z* = 867.5 [M + 2H]^2+^

^*nat*^*Y-HA-DOTATATE* (C_65_H_86_N_14_IO_19_S_2_Y): calculated monoisotopic mass = 1,646.5

Found: *m/z* = 1,647.2 [M + H]^+^, 1,669.1 [M + Na]^+^, 824.2 [M + 2H]^2+^

### Radiolabeling

#### ^68^Ga labeling

^68^Ga-labeling of DOTATATE and HA-DOTATATE (5 nmol) was carried out in a fully automated, GMP-compliant procedure using a Scintomics Gallelut^+^ module (Scintomics, Fürstenfeldbruck, Germany) [11,12]. Briefly, a ^68^Ge/^68^Ga generator with SnO_2_ matrix (iTHEMBA LABS, South Africa) was eluted with 1.0 M aq. HCl, and in order to minimize the reaction volume, a fraction of 1.25 mL containing approx. 80% of the eluted ^68^Ga activity was used for the labeling reaction. HEPES (3.7 M, 800 μL) was used to adjust the pH of the reaction mixture to pH 3. After heating to 95°C for 5 min, the reaction mixture was passed through a SPE cartridge (SepPak® C18, Waters, Eschborn, Germany). The cartridge was washed with water and the ^68^Ga-labeled peptides were eluted in a small volume of ethanol. For *in vitro* studies, the product fraction was diluted with PBS and used as such for further dilutions. For the *in vivo* biodistribution studies, the ethanol content of the product solution was evaporated *in vacuo*.

For quality control, radio-TLC was carried out using a Varian silica impregnated glass fiber TLC chromatography paper (Agilent Technologies, Waldbronn, Germany) and a 1:1 (*v*/*v*) mixture of 1 M aq. NH_4_OAc and MeOH as mobile phase. TLC strips were analyzed using a BioScan TLC analyzer (BioScan, Tucson, AZ, USA).

#### ^177^Lu labeling

For ^177^Lu-complexation, c.a. ^177^LuCl_3_ (85 MBq, approx. 20 μL, SA 1100 GBq/mg) in 0.05 M HCl (IDB Radiopharmacy bv, The Netherlands) was transferred to an Eppendorf vial, and 22.5 μL of DOTA-peptide (100 μM in water, 2.25 nmol, 4.5 molar equivalents over ^177^Lu as calculated from the specific activity at the day of the experiment) was added. The mixture was diluted with NH_4_OAc (0.1 M) to a total volume of 200 μL (pH = 5 in the final reaction mixture) and heated to 95°C for 30 min. Upon cooling to room temperature and determination of the labeling yield using Radio-TLC, the labeling mixture was directly diluted with assay buffer to a final peptide concentration of 20 nM and used as such for the subsequent *in vitro* studies.

#### Radioiodination of (Leu^8^,D-Trp^22^,Tyr^25^)-somatostatin-28 and Tyr^3^-octreotide

Radioiodination of (Leu^8^,D-Trp^22^,Tyr^25^)-somatostatin-28 and Tyr^3^-octreotide (TOC) was carried out using the IodoGen® method. Briefly, 200 μg of peptide were dissolved in 0.5 mL TRIS iodination buffer (25 mM Tris–HCl, 0.4 M NaCl, pH 7.5) and transferred to an Eppendorf reaction tube coated with 150 μg of IodoGen®. Upon addition of [^125^I]NaI (18 to 20 MBq, Hartmann Analytik, Braunschweig, Germany), the reaction vessel was briefly vortexed, and the labeling reaction was allowed to proceed for 15 min at RT. The peptide solution was then removed from the insoluble oxidizing agent. Separation of the radioiodinated peptides from unlabeled precursor was achieved using gradient RP-HPLC (gradient: 22% to 37% solvent B within 20 min, flow: 1 mL/min). For the subsequent *in vitro* studies, the respective HPLC product fraction was used as such and diluted to the required concentration using assay buffer.

### Determination of lipophilicity

Lipophilicities of [^68^Ga]DOTATATE/HA-DOTATATE and [^177^Lu]DOTATATE/HA-DOTATATE were determined via a modified shake flask method as described previously [13].

### *In vitro* studies

#### Determination of IC_50_ using cell membranes

Cell membrane preparations of cells expressing the human sst_1–5_ were obtained from Millipore (Schwalbach, Germany) and PerkinElmer (Rodgau, Germany). Competition binding studies were performed in close analogy to the manufacturer’s protocol. Briefly, solutions of unlabeled competitor (^nat^Ga-DOTATATE, ^nat^Lu-DOTATATE, ^nat^Ga-HA-DOTATATE, ^nat^Lu-HA-DOTATATE; 10 μL, concentration range from 1 × 10^−11^ to 1 × 10^−4^ M, *n* = 3 per concentration and peptide) in binding buffer (50 mM HEPES, 5 mM MgCl_2_, 1 mM CaCl_2_, 0.2% BSA, buffered to pH 7.4 using 1 N NaOH) were pipetted into a 96-well plate. To each well, 10 μL of (Leu^8^,D-Trp^22^,[^125^I]Tyr^25^)-somatostatin-28 in binding buffer (approx. 100,000 cpm) were added. Then, 5 μg of cell membrane in 80 μL of binding buffer were added per well and incubated for 60 min at RT. The final radioligand concentration in the incubation mixture was 0.42 nM. Membranes were then aspired onto a glass fiber filtermat (Printed Filtermat B, Wallach, Turku, Finland), which had been preconditioned with 0.33% polyethylenimine for 30 min and then washed five times with 300 μL (per well) of wash buffer (50 mM HEPES, 500 mM NaCl, 0.1% BSA, buffered to pH 7.4 using 1 N NaOH), using a Mach II M Harvester 96 (Tomtec CE, Etten-Leur, The Netherlands). Membranes were washed ten times with 150 μL (per well) of wash buffer. The filtermat was then cut into squares containing the membrane from the respective wells, and membrane bound activity was counted in a γ-counter. IC_50_ values were calculated from bound activity [cpm] using PRISM 6 software (Graph Pad Software, San Diego, CA, USA).

#### Dual-tracer internalization studies using AR42J cells

AR42J cells (rat pancreatic adenocarcinoma) were obtained from ECACC (European Collection of Cell Cultures, Salisbury, UK). Cells were maintained in RPMI 1640 (Biochrom, Berlin, Germany) supplemented with 10% FCS (Seromed-Biochrom, Berlin, Germany) and 2 mM l-glutamine (Gibco BRL Life Technologies, Karlsruhe, Germany). Cells were maintained at 37°C in a 5% CO_2_/humidified air atmosphere. In the assay medium used for internalization studies, FCS was replaced by 5% BSA (Sigma-Aldrich, Munich, Germany).

To avoid the influence of inter-experimental variations in cell count and cell viability on absolute internalization data, all internalization experiments were performed as dual-tracer studies using [^125^I]TOC as an internal reference. Experiments were carried out as previously described with minor modifications [14]. Briefly, after preconditioning of the cells (approx. 200,000 cells/well) with 200 μL of assay medium for a minimum of 15 min, 25 μL (per well) of either assay medium (total internalization) or 50 μM unlabeled TOC (non-specific internalization) were added, followed by the addition of 25 μL of assay medium containing a mixture of the respective ^68^Ga/^177^Lu-labeled DOTA-peptide and [^125^I]TOC, respectively. Final concentrations of ^68^Ga/^177^Lu-peptide and [^125^I]TOC in the assay medium were 1 nM (^68^Ga)/2 nM (^177^Lu) and 0.1 nM, respectively, and were calculated based on the specific activities of the radioligands used. After incubation at 37°C for different time points up to 60 min, the incubation medium was removed, and cells were rinsed with 250 μL of unsupplemented medium. The combined medium fractions represent the amount of free radioligand. Receptor bound (acid releasable) radioactivity was then removed using 2 × 250 μL of ice cold acid wash buffer (0.02 M NaOAc buffered with AcOH to pH = 5). The internalized activity was released by incubation with 250 μL of 1 N NaOH, transferred to vials and combined with 250 μL of PBS used for rinsing the wells. Quantification of the amount of free, acid-releasable, and internalized activity was performed in a γ-counter. Data for total internalized ligand (mean ± SD) were corrected by non-specific internalization (mean ± SD) at the respective time point using Excel considering the laws of error propagation.

### Biodistribution experiments

All animal experiments were performed in accord with current animal welfare regulations in Germany (approval #55.2-1-54-2532-71-13).

#### AR42J tumor model

To establish tumor growth, AR42J cells were detached from the surface of the culture flasks using 1 mM EDTA in PBS, centrifuged, and resuspended in serum-free culture medium. Concentration of the cell suspension was 2.5 to 5 × 10^6^ cells/100 μL. Nude mice (CD-1 nu/nu, female, 6 to 8 weeks, from Charles River WIGA GmbH, Sulzfeld, Germany) were injected with 100 μL of the cell suspension subcutaneously into the flank. Ten days after tumor transplantation, all mice showed solid palpable tumor masses (tumor weight 20 to 90 mg) and were used for the experiments.

#### Biodistribution studies

The ^68^Ga-labeled peptides, 1.1 to 1.7 MBq (depending on the respective specific activity of the radiotracer; the peptide amount per mouse was kept constant at 25 pmol) in 100 μL of PBS (pH 7.4), were injected i.v. into the tail vein of nude mice bearing an AR42J tumor. For competition studies, 20 μg TOC (0.8 mg/kg) were coinjected with the radioligands. The animals (groups of 4 to 5) were sacrificed 1 h post injection and the organs of interest were dissected. The radioactivity was measured in weighted tissue samples using a γ-counter. Data are given in %iD/g and are means ± SD.

## Results and discussion

### Radiolabeling

The synthesis of [^68^Ga]DOTATATE and [^68^Ga]HA-DOTATATE was performed according to an optimized protocol, yielding both tracers in >99% radiochemical purity and specific activities (SA) of 62 ± 11 and 68 ± 10 GBq/μmol, respectively. [^177^Lu]HA-DOTATATE was obtained in the same radiochemical purity but with a lower SA of 38 GBq/μmol due to the comparably low specific activity of the ^177^Lu used.

Radioiodination via the IodoGen® method afforded the reference ligands [^125^I]TOC and [Leu^8^,D-Trp^22^,[^125^I]Tyr^25^]-somatostatin 28 in radiochemical purities >99% and yields of 68 ± 4% and 54 ± 5% after HPLC purification, respectively. Since the HPLC conditions applied allowed very efficient separation of the radioiodinated products from the unlabeled precursors (Δ*t*_R_ ≥ 5 min) and no co-eluting carrier peak was observed in the labeled peptide peak in the quality control UV chromatograms, the specific activity of [^125^I]TOC and [Leu^8^,D-Trp^22^,[^125^I]Tyr^25^]-somatostatin 28 was assumed to be that of the radioiodide used for their preparation (≥74 GBq/μmol).

### Lipophilicity

The lipophilicities of the respective ^68^Ga- and ^177^Lu- complexes of DOTATATE and HA-DOTATATE are summarized in Table 1. As expected, iodine-for-hydrogen substitution in Tyr^3^ leads to an increase in lipophilicity of the [^68^Ga/^177^Lu]HA-DOTATATE analogs compared to their respective DOTATATE counterparts. Furthermore, both ^177^Lu-labeled compounds display an enhanced lipophilicity compared to their corresponding ^68^Ga analogs. This was also anticipated based on the documented different geometries of the ^68^Ga-DOTA and the ^177^Lu-DOTA chelates. While all carboxylate pendant arms of DOTA (in addition to the DOTA-peptide-amide bond) are involved in the ^177^Lu-DOTA complex for complete saturation of the coordination sphere of ^177^Lu, one of the carboxylate pendant arms is free in the ^68^Ga-DOTA complexes [15].

**Table 1:** **Lipophilicities of [** ^**68**^ **Ga]DOTATATE and [** ^**68**^ **Ga]HA-DOTATATE and their respective** ^**177**^ **Lu-labeled analogs**

**Peptide**	**log** ***P***_**O/PBS**_
[^68^Ga]DOTATATE	−3.69
[^177^Lu]DOTATATE	−3.16
[^68^Ga]HA-DOTATATE	−3.12
[^177^Lu]HA-DOTATATE	−2.69

Partition coefficients (log*P*_O/PBS_) of the radioligands between PBS (pH 7.4) and *n*-octanol were determined using a shake flask method (*n* = 6) [13].

### *In vitro* studies

#### IC_50_ studies using membrane preparations (sst_1–5_)

Affinities (IC_50_ in nM) of the respective ^nat^Ga and ^nat^Lu complexes of DOTATATE and HA-DOTATATE as well as ^nat^Y-HA-DOTATATE for hsst_1–5_ are summarized in Table 2. Data were determined using membrane preparations of CHO cells stably transfected with the respective sst subtype. Since in our earlier studies [7,8] only preliminary results (*n* = 2 experiments) had been published, further experiments were performed to validate these data.

**Table 2:** **Affinity profiles of the** ^**nat**^ **Ga,** ^**nat**^ **Lu, and** ^**nat**^ **Y complexes of DOTATATE and HA-DOTATATE for hsst**_**1**_ **-hsst**_**5**_ **receptors**

**Peptide**	**hsst**_**1**_	**hsst**_**2**_	**hsst**_**3**_	**hsst**_**4**_	**hsst**_**5**_
^nat^Ga-DOTATATE	>1,000 (2)	1.2 ± 0.6 (5)	>1,000 (2)	>1,000 (3)	>1,000 (3)
^nat^Lu-DOTATATE	>1,000 (2)	2.0 ± 0.8 (5)	162 ± 16 (2)	>1,000 (3)	>1,000 (3)
^nat^Ga-HA-DOTATATE	>1,000 (2)	1.4 ± 0.8 (5)	>1,000 (2)	>1,000 (3)	102 ± 65 (4)
^nat^Lu-HA-DOTATATE	>1,000 (2)	2.0 ± 1.6 (5)	93 ± 1 (2)	>1,000 (3)	222 ± 148 (4)
^nat^Y-HA-DOTATATE	n.d.	2.4 ± 0.3 (2)	n.d.	n.d.	680 (1)

Affinity profiles were determined using membrane preparations of CHO cells stably transfected with the respective sst subtype (supplier: Millipore) and [Leu^8^,D-Trp^22^,[^125^I]Tyr^25^]-somatostatin 28 as the radioligand. Values represent IC_50_ ± SD [nM]; the number of independent experiments is shown in parentheses. n.d., not determined.

In comparison to the previously published affinities, absolute IC_50_ values are slightly increased when averaging the full data set. However, it was confirmed that ^nat^Ga-DOTATATE and ^nat^Ga-HA-DOTATATE as well as the corresponding ^nat^Lu pair, respectively, have identical and high hsst_2_ affinities, with slightly lower affinities of the ^nat^Lu-peptides (as well as ^nat^Y-HA-DOTATATE) compared to the corresponding ^nat^Ga analogs. This observation is in agreement with previous studies by Reubi et al. [16] and Antunes et al. [4] that demonstrated a generally enhanced sst_2_ affinity of various sst-targeted ^nat^Ga-DOTA-octapeptides over their respective ^nat^Lu/^nat^Y/^nat^In analogs. It is important to note, however, that the absolute IC_50_ values obtained in the present study using membrane preparations (Table 2) are substantially higher than those obtained by Reubi et al. for ^nat^Ga-DOTATATE and ^nat^Y-DOTATATE using receptor autoradiography (0.20 ± 0.04 and 1.6 ± 0.4 nM, respectively) [16]. The reduced ‘sensitivity’ of our experimental setup might also explain why ^nat^Ga-DOTATATE and ^nat^Lu-DOTATATE showed virtually no hsst_4_ and hsst_5_ affinity in this study, while affinities in the range of 200 to 500 nM were observed in the autoradiography assay [16].

In this context, the confirmed substantially enhanced hsst_5_ affinities of ^nat^Ga-/^nat^Lu-HA-DOTATATE compared to the respective DOTATATE analogs, which represents the rationale for terming these compounds ‘high-affinity DOTATATE’ (HA-DOTATATE), are even more noteworthy.

Unfortunately, at the time of the second series of experiments, the hsst_3_ ChemiSCREEN® membrane (Millipore, Schwalbach, Germany) preparation was not available from the manufacturer anymore, and therefore, the data set for this sst subtype could not be completed under the original experimental conditions. To nevertheless be able to fully evaluate the sst subtype specificities of ^nat^Ga-/^nat^Lu-HA-DOTATATE, membrane preparations from CHO-K1 cells transfected with hsst_2–5_ were purchased from an alternative supplier (PerkinElmer), and experiments were repeated under experimental conditions identical to the previous experiments in triplicate. Unexpectedly, absolute IC_50_ values were found to be generally increased by a factor of 2.5 to 3.5 compared to the values obtained using the ChemiSCREEN® membranes, independently of the hsst subtype assayed. For example, hsst_2A_ affinities determined for ^nat^Ga- and ^nat^Lu-DOTATATE and ^nat^Ga-, ^nat^Lu-, and ^nat^Y-HA-DOTATATE in this assay were 2.9 ± 0.6, 7.3 ± 2.6, 3.8 ± 1.0, 6.6 ± 1.4, and 6.7 ± 0.1 nM, respectively. Despite the upward shift in absolute IC_50_ values, these data very well reflect the relative affinities determined using the ChemiSCREEN® membranes. The same was also observed for the data obtained for hsst_3–5_.

Thus, despite the limited numerical compatibility of the two separate data sets, both satisfactorily reflect the important differences in the sst subtype affinity profiles of the different DOTATATE and HA-DOTATATE analogs, namely the substantially enhanced hsst_5_ (and hsst_3_) affinities of the HA-DOTATATE compounds. Therefore, the data in Table 2 were deemed satisfactorily significant to serve as representative values.

#### Dual-tracer internalization studies

Comparative internalization studies for [^68^Ga]DOTATATE, [^68^Ga]HA-DOTATATE and [^177^Lu]HA-DOTATATE were carried out using AR42J rat pancreatic carcinoma cells (RPMI-1640 medium (5% BSA), 37°C). To eliminate the influence of inter-experimental variations in cell count and cell viability on absolute tracer uptake, all studies were carried out as dual-tracer studies using [^125^I]TOC as an internal reference. Radioligand concentrations were 1 nM for the ^68^Ga-labeled compounds, 2 nM for [^177^Lu]HA-DOTATATE (to ensure acceptable count rates despite lower specific activity) and 0.1 nM for [^125^I]TOC in all experiments. To ensure accurate data normalization, the same [^125^I]TOC preparation was used for all experiments in this series.

**Figure 2:**
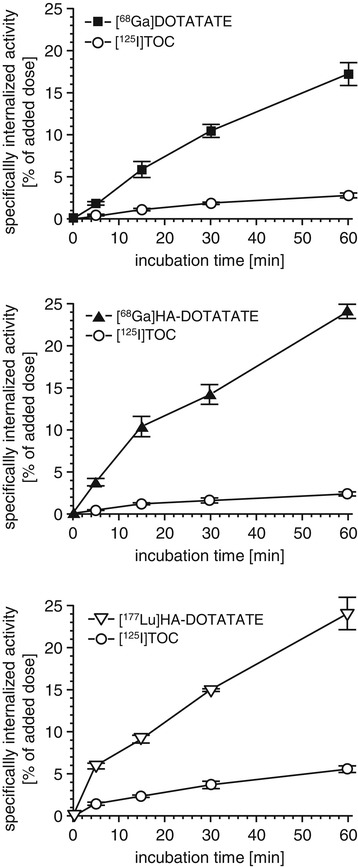
**Internalization of [** ^**68**^ **Ga]DOTATATE, [** ^**68**^ **Ga]HA-DOTATATE, [** ^**177**^ **Lu]HA-DOTATATE, and the internal reference [** ^**125**^ **I]TOC into AR42J cells.** Data are means ± SD (*n* = 3) and are corrected for non-specific internalization in the presence of 5 μM unlabeled TOC.

An improved internalization efficiency of radiolabeled TATE analogs compared to the respective TOC or OC analogs is well documented throughout the literature [14,17,18]. Thus, as expected, all radiometallated TATE analogs in this study showed significantly enhanced internalization compared to the internal standard [^125^I]TOC. At all time points, internalization efficiency was highest for [^68^Ga]HA-DOTATATE (1,000 ± 113% of [^125^I]TOC), followed by [^68^Ga]DOTATATE (570 ± 49% of [^125^I]TOC) and [^177^Lu]HA-DOTATATE (425 ± 45% of [^125^I]TOC). Interestingly, the difference in internalization efficiency between ^68^Ga- and ^177^Lu-labeled HA-DOTATATE nicely reflects the difference in sst_2_ affinity of the compounds (Table 2) and is in accordance with results from the literature [4]. In contrast, the almost doubled internalization of [^68^Ga]HA-DOTATATE compared to [^68^Ga]DOTATATE was unexpected, considering their identical sst_2_ affinities. However, it has already been demonstrated that internalization efficiency of TATE analogs does not necessarily correlate with sst_2_ affinity [14,19]. Furthermore, the fact that the sst_2_ affinities were determined in membrane preparations expressing the human receptor, while internalization studies were carried out using rat-sst_2_-expressing AR42J cells, might also contribute to the discrepancies observed between binding and internalization data (Figure 2).

### Biodistribution studies

Biodistribution data for [^68^Ga]DOTATATE and [^68^Ga]HA-DOTATATE in AR42J tumor-bearing nude mice at 60 min post injection (p.i.) are summarized in Table 3.

**Table 3:** **Biodistribution of [** ^**68**^ **Ga]DOTATATE and [** ^**68**^ **Ga]HA-DOTATATE in AR42J tumor-bearing nude mice at 60 min p.i.**

	**[** ^**68**^ **Ga]DOTATATE**		**[** ^**68**^ **Ga]HA-DOTATATE**	
	**Control**	**Competition**	**Control**	**Competition**
Blood	0.3 ± 0.06	0.4 ± 0.2	0.7 ± 0.3	2.5 ± 0.6
Heart	0.3 ± 0.04	0.2 ± 0.1	0.5 ± 0.2	1.0 ± 0.2
Lung	8.4 ± 1.4	0.6 ± 0.2	13.2 ± 1.0	2.5 ± 0.4
Liver	0.5 ± 0.1	0.2 ± 0.05	1.0 ± 0.2	1.0 ± 0.3
Intestine	2.7 ± 0.8	0.6 ± 0.2	4.0 ± 0.7	0.8 ± 0.04
Kidney	3.9 ± 0.5	4.1 ± 0.9	5.3 ± 1.0	17.1 ± 8.7
Spleen	0.9 ± 0.1	0.2 ± 0.03	1.9 ± 0.8	0.7 ± 0.2
Muscle	0.3 ± 0.2	0.2 ± 0.1	0.2 ± 0.1	0.6 ± 0.4
Stomach	15.9 ± 3.9	0.7 ± 0.1	21.7 ± 4.5	1.9 ± 0.8
Adrenals	5.1 ± 1.4	0.4 ± 0.1	10.8 ± 3.2	1.0 ± 0.3
Pancreas	26.1 ± 4.9	0.6 ± 0.03	36.6 ± 4.3	1.5 ± 0.5
AR42J tumor	24.1 ± 4.9	2.0 ± 0.1	33.6 ± 10.9	5.6 ± 0.7

For control experiments, animals (*n* = 5 per group) were injected with tracer only. For competition experiments, 20 μg/mouse of unlabeled TOC were coinjected with the respective radioligand (*n* = 3 per group). Data are expressed as %ID/g (mean ± SD).

Compared to [^68^Ga]DOTATATE, [^68^Ga]HA-DOTATATE displays a slightly delayed blood clearance, which is accompanied by an increased non-specific accumulation in the excretion organs, especially in the liver and intestine. These combined effects are most probably due to the enhanced lipophilicity of [^68^Ga]HA-DOTATATE compared to [^68^Ga]DOTATATE (Table 1), leading to both a higher fraction of plasma protein binding [20] and a slight shift of renal towards hepatobiliary excretion. Generally, the non-specific tracer distribution and excretion pattern observed for [^68^Ga]HA-DOTATATE in AR42J tumor-bearing mice fully mirror the observations made in our previously published comparative PET imaging and dosimetry studies in patients [7-9].

**Figure 3:**
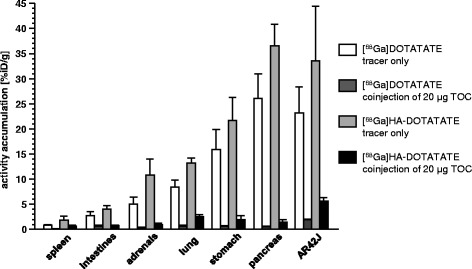
**Sst-specific tissue uptake of [** ^**68**^ **Ga]DOTATATE and [** ^**68**^ **Ga]HA-DOTATATE.** Effect of coinjection of 20 μg of unlabeled TOC per mouse (0.8 mg/kg) on the uptake of [^68^Ga]DOTATATE and [^68^Ga]HA-DOTATATE in sst-positive normal tissues and tumor of AR42J tumor-bearing nude mice at 60 min p.i. Data are means ± SD with *n* = 5 in the control experiment and *n* = 3 in the competition experiment.

Due to its enhanced internalization efficiency, [^68^Ga]HA-DOTATATE shows high uptake in sst-expressing mouse tissues such as the stomach, adrenals, pancreas, and the AR42J tumor xenografts (increased by a factor of 1.4 to 2 compared to [^68^Ga]DOTATATE) [21]. Sst specificity of tracer uptake in these organs was confirmed in a competition study by coinjection of an excess of unlabeled TOC (Table 3 and Figure 3). Interestingly, sst-mediated tracer accumulation was also observed in the lung, spleen, and intestines both for [^68^Ga]DOTATATE and [^68^Ga]HA-DOTATATE, underlining the suitability of both radioligands for the sensitive *in vivo* detection of even low sst expression levels. Of the two compounds investigated, [^68^Ga]HA-DOTATATE showed a slightly higher proportion of non-blockable tissue uptake in the competition study, reflecting the higher blood activity concentration of this compound under the experimental conditions.

**Figure 4:**
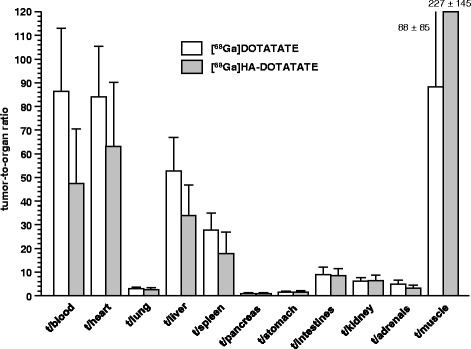
**Tumor-to-organ ratios of [** ^**68**^ **Ga]DOTATATE and [** ^**68**^ **Ga]HA-DOTATATE.** Biodistribution experiments were carried out in AR42J tumor-bearing nude mice (60 min p.i.). Data are means ± SD (*n* = 5).

Again, the preclinical mouse data obtained in this study parallel the findings from our human PET studies, where [^68^Ga]HA-DOTATATE showed somewhat improved sst-targeting efficiency compared to [^68^Ga]DOTATATE, reflected by a slight increase in SUV_mean_ for the spleen and pituitary [8]. However, while the mouse biodistribution data in this study reveal fundamental differences between both the general biodistribution and sst-mediated tissue accumulation of [^68^Ga]HA-DOTATATE and [^68^Ga]DOTATATE, respectively, these opposed effects were not observed to the same extent in the human studies. For example, tracer uptake (SUV_mean_) in normal organs such as the liver and kidney as well as in primary tumors and their metastases were found to be nearly identical for both radioligands in patients [8]. Obviously, tissue distribution of [^68^Ga]HA-DOTATATE and [^68^Ga]DOTATATE in humans is mainly dominated by the very similar tracer pharmacokinetics of both compounds, whereas in mice, the respective influence of both the increased sst-targeting efficiency and of the slightly enhanced lipophilicity of [^68^Ga]HA-DOTATATE on tracer biodistribution is much more pronounced. Interestingly, the comparable *in vivo* performance of [^68^Ga]HA-DOTATATE and [^68^Ga]DOTATATE in patients is well reflected by the very similar tumor-to-background ratios (Figure 4) obtained for the two compounds in the AR42J xenograft model. Here, the high sst-targeting efficiency of [^68^Ga]HA-DOTATATE is counterbalanced by the enhanced tracer accumulation in non-target organs occasioned by the delayed clearance kinetics of [^68^Ga]HA-DOTATATE. It is important to note that, given the early time point after tracer injection (1 h p.i.), these data certainly do not display the full sst-targeting potential of [^68^Ga]HA-DOTATATE. Its increased blood activity levels at 1 h p.i. may lead to improved bioavailability and even higher tumor uptake at later time points, which might prove advantageous for PRRT using [^177^Lu]HA-DOTATATE. However, longer circulation times also might entail increased hematotoxicity, and therefore, it needs to be carefully evaluated in preclinical therapeutic studies, to what extent the differences between [^177^Lu]DOTATATE and [^177^Lu]HA-DOTATATE will have an impact on the therapeutic effects of the compounds.

## Conclusions

The previous observations from our patient studies demonstrating nearly identical performance of [^68^Ga]HA-DOTATATE and [^68^Ga]DOTATATE in PET have now been complemented and confirmed by detailed comparative preclinical results. [^68^Ga]HA-DOTATATE shows enhanced internalization *in vitro* and at least equal *in vivo* tumor targeting compared to [^68^Ga]DOTATATE. Based on unlimited precursor availability, [^68^Ga]HA-DOTATATE therefore represents a useful alternative to other currently used ^68^Ga-labeled somatostatin analogs and, as suggested by affinity and internalization data, may have potential for targeted radiotherapy using, e.g., ^177^Lu. To assess its suitability for this application, however, careful evaluation taking into account the slightly altered pharmacokinetics of HA-DOTATATE compared to DOTATATE and the potential risks associated therewith is warranted.

## References

[1] Mojtahedi A, Thamake S, Tworowska I, Ranganathan D, Delpassand ES (2014). The value of (68)Ga-DOTATATE PET/CT in diagnosis and management of neuroendocrine tumors compared to current FDA approved imaging modalities: a review of literature. Am J Nucl Med Mol Imaging..

[2] Banerjee SR, Pomper MG (2013). Clinical applications of gallium-68. Appl Radiat Isotopes..

[3] Velikyan I, Sundin A, Sorensen J, Lubberink M, Sandstrom M, Garske-Roman U (2014). Quantitative and qualitative intrapatient comparison of Ga-68-DOTATOC and Ga-68-DOTATATE: net uptake rate for accurate quantification. J Nucl Med..

[4] Antunes P, Ginj M, Zhang H, Waser B, Baum RP, Reubi JC (2007). Are radiogallium-labelled DOTA-conjugated somatostatin analogues superior to those labelled with other radiometals?. Eur J Nucl Med Mol I..

[5] Wild D, Bomanji JB, Benkert P, Maecke H, Ell PJ, Reubi JC (2013). Comparison of Ga-68-DOTANOC and Ga-68-DOTATATE PET/CT within patients with gastroenteropancreatic neuroendocrine tumors. J Nucl Med..

[6] Schuchardt C, Kulkarni HR, Prasad V, Zachert C, Muller D, Baum RP (2013). The Bad Berka dose protocol: comparative results of dosimetry in peptide receptor radionuclide therapy using (177)Lu-DOTATATE, (177)Lu-DOTANOC, and (177)Lu-DOTATOC. Recent Results Cancer Res..

[7] Brogsitter C, Schottelius M, Zophel K, Kotzerke J, Wester HJ (2013). Twins in spirit: DOTATATE and high-affinity DOTATATE. Eur J Nucl Med Mol I..

[8] Brogsitter C, Zophel K, Hartmann H, Schottelius M, Wester HJ, Kotzerke J (2014). Twins in spirit part II: DOTATATE and high-affinity DOTATATE-the clinical experience. Eur J Nucl Med Mol I..

[9] Hartmann H, Freudenberg R, Oehme L, Zophel K, Schottelius M, Wester HJ (2014). Dosimetric measurements of 68Ga-high affinity DOTATATE. Twins in spirit - part III. Nuklearmedizin..

[10] Schottelius M, Schwaiger M, Wester HJ (2003). Rapid and high-yield solution-phase synthesis of DOTA-Tyr(3)-octreotide and DOTA-Tyr(3)-octreotate using unprotected DOTA. Tetrahedron Lett..

[11] Notni J, Simecek J, Hermann P, Wester HJ (2011). TRAP, a powerful and versatile framework for gallium-68 radiopharmaceuticals. Chem-Eur J..

[12] Notni J, Pohle K, Wester HJ (2012). Comparative gallium-68 labeling of TRAP-, NOTA-, and DOTA-peptides: practical consequences for the future of gallium-68-PET. EJNMMI Res..

[13] Schottelius M, Wester HJ, Reubi JC, Senekowitsch-Schmidtke R, Schwaiger M (2002). Improvement of pharmacokinetics of radioiodinated Tyr(3)-octreotide by conjugation with carbohydrates. Bioconjugate Chem..

[14] Schottelius M, Reubi JC, Eltschinger V, Schwaiger M, Wester HJ (2005). N-terminal sugar conjugation and C-terminal Thr-for-Thr(ol) exchange in radioiodinated Tyr(3)-octreotide: effect on cellular ligand trafficking in vitro and tumor accumulation in vivo. J Med Chem..

[15] Viola-Villegas N, Doyle RP (2009). The coordination chemistry of 1,4,7,10-tetraazacyclododecane-N, N ', N '', N '''-tetraacetic acid (H(4)DOTA): structural overview and analyses on structure-stability relationships. Coordin Chem Rev..

[16] Reubi JC, Schar JC, Waser B, Wenger S, Heppeler A, Schmitt JS (2000). Affinity profiles for human somatostatin receptor subtypes SST1-SST5 of somatostatin radiotracers selected for scintigraphic and radiotherapeutic use. Eur J Nucl Med..

[17] de Jong M, Breeman WAP, Bakker WH, Kooij PPM, Bernard BF, Hofland LJ (1998). Comparison of In-111-labeled somatostatin analogues for tumor scintigraphy and radionuclide therapy. Cancer Res..

[18] Storch D, Behe M, Walter MA, Chen JH, Powell P, Mikolajczak R (2005). Evaluation of [Tc-99 m/EDDA/HYNIC0]octreotide derivatives compared with [In-111-DOTA(0), Tyr(3), Thr(8)]octreotide and [In-111-DTPA(0)]octreotide: does tumor or pancreas uptake correlate with the rate of internalization?. J Nucl Med..

[19] Ginj M, Chen JH, Walter MA, Eltschinger V, Reubi JC, Maecke HR (2005). Preclinical evaluation of new and highly potent analogues of octreotide for predictive imaging and targeted radiotherapy. Clin Cancer Res..

[20] Kratochwil NA, Huber W, Muller F, Kansy M, Gerber PR (2002). Predicting plasma protein binding of drugs: a new approach. Biochem Pharmacol..

[21] Kraus J, Woltje M, Schonwetter N, Hollt V (1998). Alternative promoter usage and tissue specific expression of the mouse somatostatin receptor 2 gene. Febs Lett..

